# Clinical features of severe fever with thrombocytopenia syndrome and analysis of risk factors for mortality

**DOI:** 10.1186/s12879-021-06946-3

**Published:** 2021-12-14

**Authors:** Feng He, Xinxin Zheng, Zhaoru Zhang

**Affiliations:** Department of Infectious Diseases, Chaohu Hospital Affiliated With Anhui Medical University, Hefei, China

**Keywords:** Fever with thrombocytopenia, Clinical progression, Mortality, Prediction

## Abstract

**Background:**

To understand the clinical characteristics of and explore the risk factors for mortality in patients with severe fever with thrombocytopenia syndrome (SFTS).

**Methods:**

Data from SFTS patients diagnosed by laboratory examination at Chaohu Hospital affiliated with Anhui Medical University from June 2017 to January 2021 were retrospectively analysed. According to the clinical results, all confirmed patients were divided into the surviving group (80 patients) and non-surviving group (20 patients). The two groups were compared in terms of general characteristics, clinical symptoms and signs, laboratory indicators and other aspects. The independent risk factors for mortality in SFTS patients were analysed by multivariate binary logistic regression.

**Results:**

Univariate analysis showed a significant difference in age and the incidence of consciousness disturbance, respiratory failure, haemorrhagic manifestations, renal dysfunction, shock, aspartate aminotransferase (AST) ≥400 U/L, creatine kinase (CK)≥1000 U/L, creatine kinase isoenzymes (CK-MB) ≥100 U/L, lactate dehydrogenase (LDH) ≥1000 U/L, serum creatinine ≥100 mmol/L, blood urea nitrogen ≥7.5 mmol/L and C-reactive protein ≥8 mg/L between the two groups (P < 0.05).

**Conclusions:**

Consciousness disorder, haemorrhagic manifestations, renal dysfunction, AST ≥ 400 U/L, and LDH ≥ 1000 U/L are independent risk factors for mortality in SFTS patients and merit close attention in clinical treatment to avoid fatal consequences.

## Background

Severe fever with thrombocytopenia syndrome (SFTS) is an emerging infectious disease caused by SFTS virus (SFTSV), a novel bunyavirus. According to the nomenclature by the International Committee on Taxonoy of Viruses (ICTV), SFTSV has been classified into the Genus Banyangvirus, Family Phenuivirdae and named as Huaiyangshan banyangvirus [1]. The disease is mainly transmitted through the bite of *Haemaphysalis longicornis* [2]. Human-to-human transmission also occurs through contact with the bodily fluid of an infected person [3]. This disease was first reported in 2010 in China [4], followed by similar infections in South Korea, Japan, Vietnam, Myanmar, Taiwan and Thailand, indicating that the distribution of SFTSV in Southeast Asia might be much more extensive than expected [5–8]. The main features of this disease are high fever with thrombocytopenia, symptoms of systemic infection, and multiple organ dysfunction, with a mortality rate of 12–30% [9, 10]. However, no licensed vaccine or pharmaceutical options are currently approved. Thus, determining the related risk factors for death and intervening early are important for reducing mortality in such patients. This study retrospectively analysed the clinical data of patients with SFTS confirmed by laboratory tests at our hospital. Our aim was to determine the risk factors for mortality in patients with SFTS and to reduce the mortality rate of SFTS through early intervention.

## Methods

### Study settings and patients

A total of 100 patients with SFTS admitted to Chaohu Hospital Affiliated With Anhui Medical University from June 2017 to January 2021 and confirmed by laboratory examination were enrolled. The inclusion criteria were as follows: (1) treatment conformed to the recommended standards in the Guidelines for the Prevention and Treatment of Severe Fever with Thrombocytopenia Syndrome (2010 Edition) [11] issued by the Ministry of Health of the People's Republic of China; and (2) a comprehensive physical and laboratory examination was performed after admission, and all data were available. The exclusion criteria were as follows: (1) overseas living history within 6 months before onset; (2) history of blood system disease prior to onset; and (3) self-discharge of patients within 72 h. According to the clinical results, all confirmed patients were divided into the surviving and non-surviving group.

After admission, all patients were treated in accordance with the Guidelines for the Prevention and Treatment of Severe Fever with Thrombocytopenia Syndrome (2010 Edition) issued by the Ministry of Health of the People's Republic of China [11]. Ribavirin (0.5 g/d) was administered to all patients from admission until the body temperature returned to normal. Plasma and platelet (PLT) transfusions were administered to patients with severe bleeding or those in thrombocytopenia crisis. For patients with severe neutropenia, recombinant human granulocyte colony-stimulating factor was administered subcutaneously. Symptomatic treatment was provided for patients with liver and kidney dysfunction and other systemic complications.

### Study design

The medical records of patients with SFTS were reviewed, the basic information, symptoms, signs and laboratory examination results of patients in the surviving group and non-surviving group were compared, and the risk factors for mortality were analysed.

### Statistical analysis

The database was established using EpiData 3.0 software. The data were entered into the database by two investigators and then compared. Measurement data with a normal distribution are expressed as X ± S, and comparisons between groups were performed by two independent samples t tests. Measurement data with a nonnormal distribution are represented by quantile intervals, and the Wilcoxon rank-sum test of two independent samples was used for comparisons between groups. In the data analysis, the normal value or a multiple of the normal value was taken as the cut-off point, and continuous variables were converted to categorical variables. Enumeration data are presented as the number of cases and percentage, and comparisons between groups were performed by the Chi-square test. Risk factors for mortality were analysed by multivariate binary logistic regression. All statistical analyses were performed using SPSS 22.0 software, and statistical significance was set at p < 0.05.

## Results

### General information

A total of 100 patients were enrolled in this study, including 46 males and 54 females. According to the clinical results, the patients were divided into the surviving group (n = 80) and the non-surviving group (n = 20). The mean age of the patients was 65.20 ± 9.86 years. Of the 100 patients, 77 (77%) lived in rural areas, and 76 (76%) were farmers. Fifty-five patients (55%) had hypertension, coronary heart disease, chronic hepatitis and other basic diseases at admission. Thirty-six patients (36%) had a clear history of tick bites prior to admission. The median time from symptom onset to hospitalization for all patients was 7 days (5–8 days). In the non-surviving group, all deaths occurred between 16 and 19 days after admission. In the surviving group, the median time from onset to discharge was 20 days (16–23 days). When general information was compared between the two groups, only age showed a statistically significant difference (P < 0.05), with no statistically significant differences in other indicators (Table 1).

**Table 1:** Comparison of general characteristics in the two study groups

General information	Total (n = 100)	Non-fatal (n = 80)	Fatal (n = 20)	Statistical analysis value	p value
Age, years	65.20 ± 9.86	64.05 ± 9.74	69.03 ± 10.18	T = -2.137	0.035
Residence, Rural/Urban	77/23 (77%/23%)	62/18 (77.5%/22.5%)	15/ 5(75%/25%)	x^2^ = 0.056	0.812
Occupations, Formers/Others	76/24 (76%/24%)	61/19 (76.3%/23.7%)	15/5 (75%/25%)	x^2^ = 0.014	0.907
Underlying diseases, n	55 (55%)	42 (52.5%)	13 (65%)	x^2^ = 1.218	0.270
Time from onset to admission, days	7 (5–8)	5 (5–7)	7 (4–8)	Z = -1.316	0.188
History of tick bite, n	36 (36%)	29 (36.3%)	7 (35%)	x^2^ = 0.011	0.917

### Clinical symptoms and signs

Among all the patients, the most common clinical symptom was fever in 98 cases (98%), and more than half of the patients had a high fever, with a body temperature over 39℃. Other common symptoms and signs were anorexia (80 cases, 80%), fatigue (68 cases, 68%) and abnormal liver function (60 cases, 60%). The 15 clinical symptoms and signs in the two groups of patients were compared and analysed. The results showed that there were statistically significant differences in the incidence of consciousness disorder, respiratory failure, haemorrhagic manifestations, renal dysfunction and shock between the two groups (P < 0.05). Disturbance of consciousness, i.e., drowsiness, confusion, lethargy, or a severe disturbance of consciousness (no Glasgow Coma Score evaluation was done), and 13 patients (65%) in the non-surviving group had consciousness disturbance. Haemorrhagic manifestations included skin ecchymosis, oral gingival bleeding, gastrointestinal bleeding, and pulmonary bleeding, and 13 patients (65%) in the non-surviving group had haemorrhagic manifestations. In the non-surviving group, 4 patients (20%) suffered from respiratory failure, and 13 patients (65%) suffered from shock. In addition, 18 patients (90%) in the non-surviving group developed renal dysfunction, representing a significantly higher proportion than that in the surviving group (17 patients, 21.3%). There was no significant difference in fever, fatigue, muscle soreness or other indicators between the two groups (P > 0.05) (Table 2).

**Table 2:** Comparison of clinical symptoms and signs between the two groups

Symptom and sign	Total (n = 100)	Non-fatal (n = 80)	Fatal (n = 20)	Wald x^2^ value	p value	OR (95% CI)
Fever	98 (98%)	78 (97.5%)	20 (100%)	0.510	0.475	0.975 (0.941–1.010)
Fatigue	68 (68%)	53 (66.2%)	15 (75%)	0.563	0.453	0.654 (0.215–1.992)
Myalgia	52(52%)	43(53.8%)	9(45%)	0.491	0.484	1.420 (0.531–3.802)
Headache	22 (22%)	17 (21.3%)	5 (25%)	0.131	0.717	0.810(0.258–2.544)
Disturbance of consciousness	39 (39%)	26 (32.5%)	13 (65)	7.104	0.008	0.259 (0.092–0.727)
Cough	20(20%)	14(17.5%)	6(30%)	1.563	0.211	0.495 (0.162–1.521)
Expectoration	19 (19%)	13 (16.3%)	6 (30%)	1.966	0.161	0.453(0.147–1.396)
Respiratory failure	5 (5%)	1 (1.3%)	4 (20%)	11.842	0.001	0.051 (0.005–0.483)
Anorexia	80(80%)	63(78.8%)	17(85%)	0.391	0.532	0.654 (0.171–2.496)
Vomiting	27 (27%)	21 (26.3%)	6 (30%)	0.114	0.735	0.831 (0.283–2.441)
Diarrhea	24 (24%)	19 (23.8%)	5 (25%)	0.014	0.907	0.934 (0.300–2.909)
Hemorrhagic manifestation	20 (20%)	7 (8.8%)	13(65%)	31.641	< 0.001	0.052 (0.016–0.172)
Liver dysfunction	60 (60%)	53 (66.3%)	17 (85%)	2.679	0.102	0.346 (0.093–1.286)
Kidney dysfunction	35 (35%)	17 (21.3%)	18 (90%)	33.242	< 0.001	0.030 (0.006–0.142)
Shock	17 (17%)	4 (5%)	13 (65%)	40.822	< 0.001	0.028 (0.007–0.111)

### Laboratory test results

**Fig 1:**
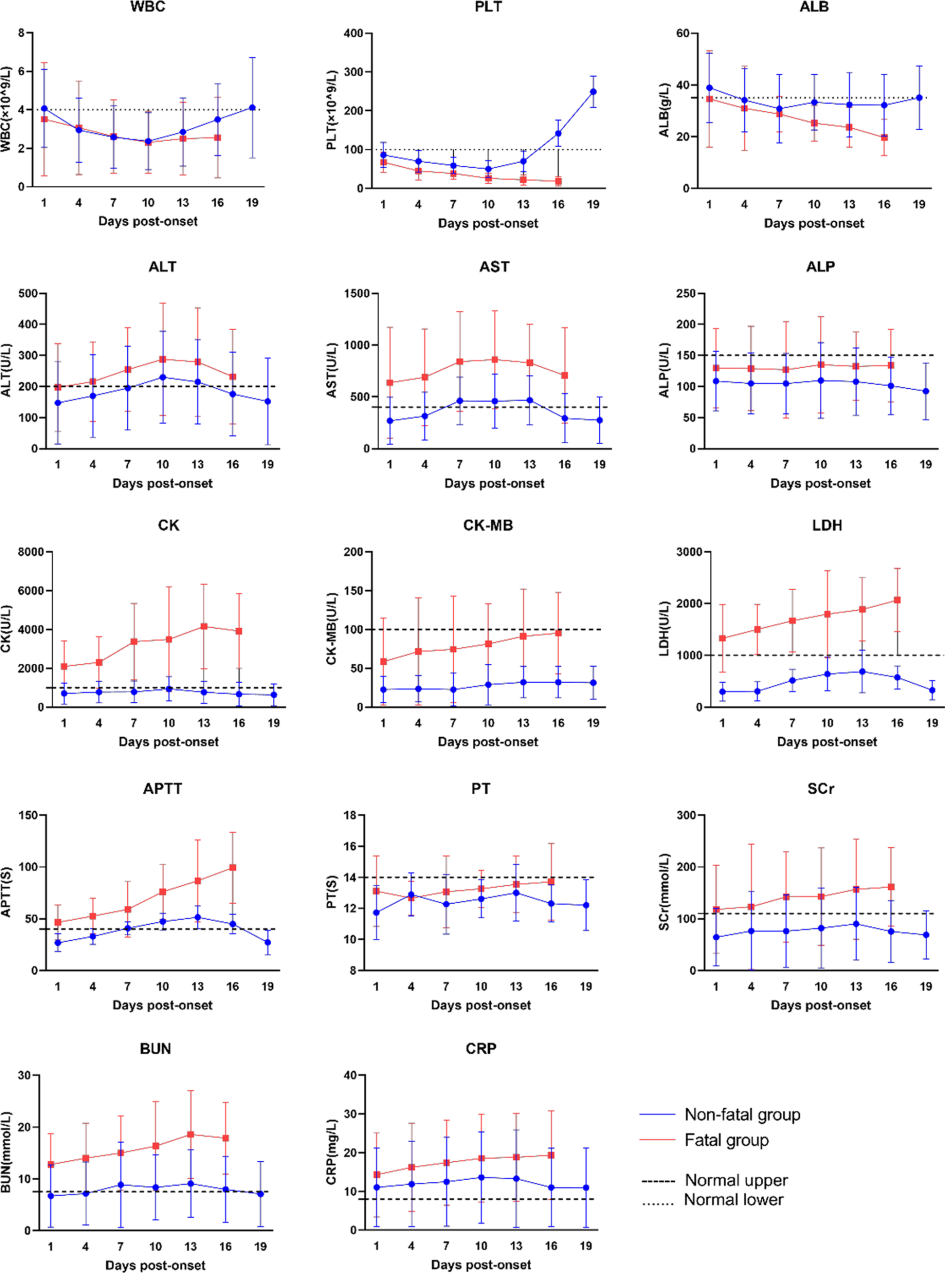
Dynamic profiles of 14 laboratory parameters in 100 SFES patients. Blue lines represent survivors and red lines indicate deceased patients. The dashed lines indicate the normal level of each parameter

To identify risk factors associated with major laboratory findings and fatal outcomes during disease progression in patients with SFTS, we performed a dynamic analysis of 14 major laboratory indicators. Patients were followed up every 3 days from day 1 to day 19 after onset. The results showed that the white blood cell (WBC) and Platelet counts of patients in the surviving group began to decrease from the onset of the disease, reached the lowest level at approximately the 10th day, and then began to increase. By the 19th day, the values recovered to near the normal levels in most patients. The WBC and Platelet counts in the non-surviving group decreased from the onset of the disease and improved slightly or did not improve after treatment. The alanine aminotransferase (ALT) and aspartate aminotransferase (AST) levels in both groups increased from onset, peaked at 7–13 days, and then decreased gradually. Most patients in the non-surviving group had a much higher AST level than those in the surviving group. The creatine kinase (CK) and lactate dehydrogenase (LDH) levels of most patients in the surviving group were within the range of normal values, increased slightly from onset, and gradually returned to normal with treatment. In the non-surviving group, the CK and LDH levels increased from onset and improved slightly or did not improve after treatment. In addition, the CK and LDH levels of most patients were far higher than the upper limit of normal and far higher than those of patients in the surviving group. In the surviving group, the activated partial thromboplastin time (APTT) increased from onset, peaked at 10–13 days, and then began to decrease, returning to near the normal level in most patients by 19 days. In the non-surviving group, the APTT increased from onset and improved slightly or not after treatment. The serum creatinine (SCr), blood urea nitrogen (BUN) and C-reactive protein (CRP) levels in the surviving group increased from onset, reached a peak on approximately the 10th day, and then decreased gradually. In the non-surviving group, the SCr, BUN and CRP levels increased from onset, reached a peak on approximately the 10th day, and then remained at the same level in most patients. Based on the above analysis, we believe that 7–13 days after the onset of SFTS is a critical period affecting the prognosis of patients, which merits attention in clinical treatment (Fig. 1).

We analysed the laboratory results of patients on day 10 and converted continuous variables to categorical variables by using the normal value or multiples of the normal value as cut-off points. The results showed that the most common laboratory test abnormality in all patients was PLT < 100 × 10^9^/L, occurring in 98 cases (98%). Other common laboratory abnormalities were APTT ≥ 40 s, occurring in 82 cases (82%), and WBC < 4 × 10^9^/L, occurring in 80 cases (80%). A comparative analysis of the 14 major laboratory tests between the two groups of patients showed that there were significant differences in the incidence of AST ≥ 400 U/L, CK ≥ 1000 U/L, CK-MB ≥ 100 U/L, LDH ≥ 1000 U/L, SCr ≥ 100 mmol/L, BUN ≥ 7.5 mmol/L and CRP ≥ 8 mg/L between the 2 groups (P < 0.05) (Table 3).

**Table 3:** Comparison of laboratory test indicators in the two study groups

Clinical manifestations	Total (n = 100)	Non-fatal (n = 80)	Fatal (n = 20)	Wald x^2^ value	p value	OR (95% CI)
WBC(< 4 × 10^9^/L)	80 (80%)	63 (78.8%)	17 (85%)	0.391	0.532	0.654 (0.171–2.496)
Platelet count(< 100 × 10^9^/L)	98 (98%)	78 (97.5%)	20 (100%)	2.025	0.155	0.975 (0.941–1.010)
ALB(< 35 g/L)	64 (64%)	49 (61.3%)	15 (75%)	1.313	0.252	0.527 (0.174–1.595)
ALT(≥ 200U/L)	32 (32%)	24(30%)	8(40%)	0.735	0.391	0.643 (0.233–1.773)
AST(≥ 400U/L)	49(49%)	31 (38.8%)	18 (90%)	16.817	< 0.001	0.070 (0.015–0.324)
ALP(≥ 150U/L)	13 (13%)	9 (11.3%)	4 (20%)	1.083	0.298	0.507(0.139–1.854)
CK(≥ 1000U/L)	36 (36%)	22 (27.5%)	14 (70%)	12.543	< 0.001	0.163 (0.055–0.476)
CK-MB(≥ 100U/L)	11(11%)	5 (6.3%)	6 (30%)	9.219	0.002	0.156 (0.042–0.580)
LDH(≥ 1000U/L)	21 (21%)	6 (7.5%)	15 (75%)	43.942	< 0.001	0.027 (0.007–0.100)
APTT(≥ 40 s)	82(82%)	63 (78.8%)	19 (95%)	2.862	0.091	0.195 (0.024–1.563)
PT(≥ 14 s)	18(18%)	13 (16.3%)	5 (40%)	0.830	0.362	0.582 (0.180–1.882)
SCr(≥ 110 mmol/L)	22 (22%)	10 (12.5%)	12 (60%)	21.037	< 0.001	0.095 (0.031–0.290)
BUN(≥ 7.5 mmol/L)	43 (43%)	27 (33.8%)	16 (80%)	13.964	< 0.001	0.127 (0.039–0.418)
CRP(≥ 8 mg/L)	62 (62%)	45 (56.3%)	17 (85%)	5.613	0.018	0.227 (0.062–0.836)

### Analysis of risk factors for mortality

Univariate analysis showed that there were significant differences in the incidence of consciousness disturbance, respiratory failure, haemorrhagic manifestations, renal dysfunction, shock, AST ≥ 400 U/L, CK ≥ 1000 U/L, CK-MB ≥ 100 U/L, LDH ≥ 1000 U/L, SCr ≥ 100 mmol/L, BUN ≥ 7.5 mmol/L, and CRP ≥ 8 mg/L between the two groups. These 12 factors were included in the multivariate binary logistic regression analysis. The results showed that consciousness disturbance, haemorrhagic manifestations, renal dysfunction, AST ≥ 400 U/L, and LDH ≥ 1000 U/L were independent risk factors for mortality in patients with SFTS (Table 4).

**Table 4:** Multivariate logistic regression analysis of risk factors for death

Variable	Coefficient (B)	p value	OR (95% CI)
Disturbance of consciousness	1.407	0.008	4.086 (1.454–11.483)
Hemorrhagic manifestation	2.964	< 0.001	19.367 (5.820–64.454)
Kidney dysfunction	3.491	< 0.001	32.824 (6.923–155.624)
AST(≥400U/L)	2.708	< 0.001	15.00 (3.250–69.230)
LDH(1000U/L)	3.611	< 0.001	37.000 (9.982–137.152)

## Discussion

As a new infectious disease, SFTS has a rapid onset and a high fatality rate. Early diagnosis and the timely treatment of symptoms and complications are crucial to improve the prognosis of patients with this disease [12]. Therefore, the early identification of risk factors associated with the severity of the disease is beneficial. We retrospectively reviewed the data of 100 patients with SFTS and systematically analysed their general characteristics, clinical symptoms and signs, laboratory parameters, and risk factors for mortality. These results provide further insight into the clinical features associated with SFTS, which can also facilitate the early identification of potentially severe or fatal cases.

In our study, patients in the non-surviving group were generally older than those in the surviving group, and advanced age was associated with mortality. This finding is consistent with that of previous reports by Guo et al. [13] and Zhan et al. [14]. This may be because older people have lower immunity to the SFTSV and are therefore more susceptible to infection and more likely to develop serious complications, potentially leading to death. SFTS can cause damage to different organs. Our study identified consciousness disturbance as an independent risk factor for mortality, which is consistent with previous reports by Gai et al. [15] and You et al. [16]. Unfortunately, the mechanism of nerve damage in this disease is still unclear. Zhao et al. [17] reported that some patients with SFTS with consciousness disorder showed typical manifestations of acute viral encephalitis in the cerebrospinal fluid, and an electrolyte imbalance may also lead to abnormal manifestations of conditions affecting the nervous system. In our study, the incidence of respiratory failure and shock in patients who progressed to death was significantly higher than that in patients who survived, and patients who progressed to death also tended to have severe multisystem impairment, which is consistent with a report by Hu et al. [18]. Through a multivariate binary logistic analysis, we found that haemorrhagic manifestations were an independent risk factor for mortality in patients with this disease; thus, it is advisable to closely observe the haemorrhagic manifestations of patients in the early stage of this disease, which is consistent with a report by Xu et al. [19]. Renal failure is also an independent risk factor for mortality from SFTS. Cui et al. [20] suggested that renal impairment usually occurs in the late stage of SFTS. Studies have confirmed that the measurement indicative of pathological lesions mainly involved the kidney. In a mouse model of SFTSV infection, the kidney is one of the main target organs [21], which also provides clues for clinical treatment.

Based on a dynamic analysis of laboratory findings in 100 patients with SFTS, we found that 7–13 days after onset of the disease is the key period for predicting the outcome of the disease. For most patients who survived this stage smoothly, clinical symptoms began to resolve, and abnormal indicators gradually returned to normal. Therefore, changes in the patient's condition at this stage should be given close attention by clinicians. Through a multivariate binary logistic regression analysis, we found that AST ≥ 400 U/L and LDH ≥ 1000 U/L were independent risk factors for mortality, which is consistent with a report by Yu et al. [22]. This may be related to the patients' liver function impairment, which is usually associated with severe liver function impairment in the late stage of SFTS in the non-surviving group. Yu et al. [22] reported that liver function impairment plays an important role in the pathogenesis of SFTS. In our study, SCr ≥ 100 mmol/L and BUN ≥ 7.5 mmol/L also had an impact on the prognosis of patients, which is consistent with a report by Cui et al. [20]. The SCr level is not significantly elevated in patients with SFTS, but it is still suggestive of renal parenchymal impairment. An elevated CRP level is also an indicator of a poor prognosis, possibly due to more severe tissue damage in these patients. Suberviola et al. [23] reported that a mild viral infection usually resulted in a slight increase or no change in the CRP level, but a severe viral infection could cause extensive tissue damage, resulting in a significant increase in the CRP level.

## Conclusion

In conclusion, if patients with SFTS have consciousness disturbance, haemorrhagic manifestations, renal dysfunction, AST ≥ 400 U/L, and LDH ≥ 1000 U/L, clinicians should be alerted and must administerearly intervention (such as immunoglobulin using and the corticosteroid impulse therapy) to prevent death. At the same time, if the patient develops respiratory failure, shock, CK ≥ 1000 U/L, CK-MB ≥ 100 U/L, SCr ≥ 100 mmol/L, BUN ≥ 7.5 mmol/L, or CRP ≥ 8 mg/L, close attention should also be given to changes in the patient's condition. Takayama et al. [12] reported that studies of potential antiviral drugs for SFTS-specific therapy have been conducted, with ribavirin and favipiravir being the most promising candidates. Calcium Channel Blockers showed good efficacy in SFTSV. Retrospective clinical data have indicated that nifedipine, one of the calcium channel blockers, reduced the case fatality rate by > fivefold. Our findings will help clinicians better understand disease progression in patients with SFTS and the key factors associated with disease severity and fatality and thus help physicians initiate supportive treatment in time to prevent rapid disease progression and thus reduce mortality from SFTS.

## Data Availability

The datasets used and/or analysed during the current study are available from the corresponding author on reasonable request.

## Abbreviations

SFTS: Severe fever with thrombocytopenia syndrome
SFTSV: Severe fever with thrombocytopenia syndrome virus
ICTV: International Committee on Taxonoy of Viruses
PLT: Platelet
WBC: White blood cell
ALT: Alanine aminotransferase
AST: Aspartate aminotransferase
CK: Creatine kinase
CK-MB: Creatine kinase isoenzymes
LDH: Lactate dehydrogenase
APTT: Activated partial thromboplastin time
SCr: Serum creatinine
BUN: Blood urea nitrogen
CRP: C-reactive protein
ALB: Albumin
ALP: Alkaline phosphatase
PT: Prothrombin time
